# Evaluation of compressive strength, microhardness and solubility of zinc-oxide eugenol cement reinforced with E-glass fibers

**DOI:** 10.1186/s12903-024-04261-2

**Published:** 2024-04-24

**Authors:** Tamer M. Hamdy

**Affiliations:** Restorative and Dental Materials Department, Oral and Dental Research Institute, National Research Centre (NRC), Giza, Dokki 12622 Egypt

**Keywords:** Zinc-oxide eugenol, ZOE, Compressive strength, Microhardness, Solubility

## Abstract

**Background:**

Zinc-oxide eugenol (ZOE) cements are among the most used temporary materials in dentistry. Although ZOE has advantages over other temporary fillers, its mechanical strength is weaker, so researchers are working to improve it. E-glass fibers have emerged as promising reinforcing fibers in recent years due to their strong mechanical behavior, adequate bonding, and acceptable aesthetics.

**Objectives:**

To evaluate and compare the compressive strength, surface microhardness, and solubility of the ZOE and those reinforced with 10 wt.% E-glass fibers.

**Methods:**

A total of 60 ZEO specimens were prepared; 30 specimens were reinforced with 10 wt.% E-glass fibers, considered modified ZOE. The characterization of the E-glass fibers was performed by XRF, SEM, and PSD. The compressive strength, surface microhardness, and solubility were evaluated. Independent sample t-tests were used to statistically assess the data and compare mean values (*P* ≤ *0.05*).

**Results:**

The results revealed that the modified ZOE showed a significantly higher mean value of compressive strength and surface microhardness while having a significantly lower mean value of solubility compared to unmodified ZOE (*P* ≤ 0.05).

**Conclusion:**

The modified ZOE with 10 wt.% E-glass fibers had the opportunity to be used as permanent filling materials.

## Background

Zinc-oxide (ZnO) powder is the main constituent of many dental products; it is mainly combined with different solutions to form dental cements such as zinc oxide eugenol, zinc polycarboxylate, and zinc phosphate dental cements. ZnO, which is a tooth-colored inorganic metal oxide particle, can induce unique properties such as biocompatibility, antibacterial effects, and durability [1, 2].

Zinc-oxide eugenol (ZOE) is an oil-based cement that has been employed in dentistry for several applications as a provisional filling material, luting agent, pulp dressing, endodontic sealer, cavity liner, and base material due to its superior marginal seal, sedative properties, and antimicrobial properties [3]. Nevertheless, a variety of adverse effects have been observed when ZOE is injected directly into the pulp because eugenol induces a chronic inflammatory response [4]. Eugenol, however, will not harm pulp tissue that has been fixed with a substance such as formocresol [5]. Pulpototomy with ZOE has been reported to be a successful capping agent for primary molars [6].

The composition of ZOE cement typically consists of a combination of eugenol bonding with 80%–90% ZnO powder. While, a eugenol bonding resin making up the remaining portion. ZOE is a kind of acid–base cement that is created by the interaction of eugenol with zinc oxide powder. The matrix of the cement is formed mainly from zinc eugenolate, which is a chelate substance. There must be at least a trace of water present for this reaction to happen. The mechanical characteristics and shape of ZOE are significantly influenced by the ZnO structure [3].

Compared to other dental cements, tissue can tolerate ZOE cements more readily, which permits a perfect sealing ability. They have antiseptic, antibacterial, sedative, and pain-relieving properties. Eugenol leaching is a contributing factor in the antibacterial and bacteriostatic actions of ZOE-based dental products [7]. Their weak mechanical qualities led to their employment as a temporary filling material. In order to improve their mechanical features and decrease their solubility, an additive could be incorporated into their composition [3].

Modification of dental materials, especially by the employment of inorganic fillers, led to great advancements in their properties [8–13]. The physical, mechanical, thermal, and tribological properties of the dental resin matrix are greatly improved by the addition of fibers [14]. Moreover, these properties are significantly influenced by its silane treatment [15]. Coupling agents enhance the bonding strength between the fillers and organic matrix [16, 17]. The characteristics of composite materials have improved to those of a simple polymer matrix [18]. Various matrixes and fillers have been combined to create a variety of current formulations [19]. It is crucial to characterize the mechanical properties of dental materials, such as their modulus, flexural strength, and compressive strength [20, 21]. By including dispersed particles or fibers, a variety of additives could be used to improve the mechanical properties of dental materials. By using a coupling agent, these particles or filaments form a strong link with the polymer matrix [22], these includes; zirconia, tricalcium phosphate, titanium oxide, aluminum oxide, and hydroxyapatite [23–29].

Glass fibers are tiny, silica-based strands with a small diameter. Glass fibers are available in a variety of compositions, including A-, C-, D-, AR-, S-, and E-glass. Although every variety of glass fiber has different characteristics and uses, they are all basically amorphous, made of a three-dimensional network of silica with oxygen and other atoms distributed at random [30].

Glass fibers have application in diverse fields such as dentistry and engineering. Their use is in the manufacturing of several dental products, such as prosthodontic, endodontic, and restorative materials [30–32]. Electric-grade glass fibers, or E-glass fibers, are the most common kind of glass fiber used in dentistry, particularly because of their great water resistance, good electrical insulation, and inexpensive cost. In addition to many benefits, including acceptable aesthetics, biocompatibility, insolubility, high flexural strength, compressive strength, and fracture toughness, to meet the unique requirements of various dental applications [33, 34]. The reinforcement of dental materials could be successfully achieved by the employment of E-glass fiber into the matrix to act as a primary load-carrying element and to protect them from harm caused by the surrounding environment [35].

The current study aimed to compare the dental ZOE modified with 10 wt.% silane-treated E-glass fiber fillers in terms of compressive strength, surface microhardness, and solubility with the ZOE (control). According to the null hypothesis, the modification of the ZOE by incorporation of 10 wt.% silane-treated E-glass fiber would not affect the compressive strength, surface microhardness, or solubility compared to the ZOE control group.

## Methods

The National Research Centre (NRC), in Cairo, Egypt's Medical Research Ethical Committee (MREC) accepted the current experimental investigation (reference number: 440542023). For this study, a commercially available ZOE was provided in powder and liquid form: Zinconol (Prevest Denpro Limited, Bari, India). The powder consists of zinc oxide, poly methyl methacrylate (PMMA) polymer and zinc acetate. While, the liquid consists of eugenol oil. Commercial micro-glass milled fiber powder was utilized as a filler (Fibertec Inc., Scotland Boulevard, Bridgewater, MA, U.S.A). The specifications of the fiber were; silane treated E-glass fiber, consists of highly transparent long aspect ratio E-glass fiberglass continuous filament, 1.6 µm diameter, 110 µm length, and 11:1 aspect ratio.

### E-glass powder analysis

#### X-ray fluorescence (XRF) spectrometry analysis

The chemical composition of the E-fiber fillers was determined by XRF (X-MET3000TXR, Oxford Instruments GmbH Co., Borsigstrasse, Germany) to identify their chemical composition [36].

#### Scanning electron microscope (SEM) analysis

The shape, particle size, and distribution of the utilized E-glass particles were investigated using an environmental scanning electron microscope (SEM) (Quanta 250 FEG, FEI Company, Hillsboro, OR, USA). The inspection was carried out at 2500X magnification, with an accelerating voltage range of 20.0 kV to 30.0 kV.

#### Particle size distribution (PSD) analysis

The E-glass particles were subjected to a particle size distribution (PSD) study using a particle sizer analysis system (PSS Nicomp 380 particle sizer, Santa Barbara, California, USA) and the dynamic light scattering technique (DLS). The average particle diameter of E-glass fiber particles was measured and conducted based on histogram analysis; Gaussian fitted distribution curves are plotted (intensity weighting, volume weighting, and number weighting).

#### Sample size calculation

The sample size calculation was based on a similar study [37]. With an alpha level of 0.05 and a power of 85%, a sample size was determined using G*Power software version 3.1.9.7 (Heinrich Heine University Duesseldorf, Duesseldorf, Germany). The minimum sample size needed with this effect size is *n* = 10 per group to test compressive strength, microhardness, and solubility.

#### Preparation of the specimens

A total of 60 specimens were prepared. Depending on the type of powder utilized for the mixing process, the specimens were divided into two main groups (*n* = 30/group).The control group was created by combining the conventional ZOE powder with their liquid; the reinforced group was created by combining 10 wt.% E-glass fiber fillers with the conventional ZOE powder. After that, their liquid was combined with the created powder. The manufacturer's instructions were followed for combining the powder and liquid. The combined material from each group was put into specially designed Teflon molds according to each test. To avoid air trapping, a polyester strip was positioned, and a glass slide gently compressed the materials on both sides of the mold. Specimens were taken out of the mold following the manufacturer's recommended setting time. After that, the specimens were visually inspected for flaws. Using 1200-grit silicon-carbide paper, all the specimens were polished to eliminate any surface flaws.

### Testing of specimens

#### Compressive strength test

Ten cylinder-shaped specimens per group (6 mm in height and 4 mm in diameter) were determined using the standard specification for Zinc oxide- eugenol cements, ISO standard 3107:2022 (Dentistry – Zinc oxide/eugenol and zinc oxide/non-eugenol cements) [38]. The specimens were removed from the moulds and kept for 24 h at 37 °C with 95 ± 5% relative humidity in an incubator (CBM, S.r.l. Medical Equipment, 2431/V, Cremona, Italy). The test was carried out in compression at a crosshead speed of 1.0 mm/min in a universal testing machine (Shimadzu Autograph AG–X Plus, Kyoto, Japan) until a fracture occurred [38].

#### Microhardness test

For each group, ten disc-shaped specimens measuring 5 mm in height and 2 mm in diameter were made. The samples were removed from the molds and left to incubate for a whole day at 37 °C in a very humid environment. A surface microhardness test was conducted using a Vickers hardness (VH) tester (NEXUS 400TM, INNOVATEST, model no. 4503, Maastricht, Netherlands). At a load of 100 g and 20 × magnification, the indentations were created in 15 s of dwell time. Vickers hardness numbers (VHN) were used to express the mean surface microhardness value for each specimen [3].

#### Solubility test

Solubility was investigated using a cylindrical polytetrafluoroethylene mold measuring 7.75 mm in diameter and 1.5 mm in thickness [39, 40], to obtain a disc-shaped specimen (*n* = 10). The specimens from each group were incubated in an incubator at 37 °C for 24 h. To get the initial mass (M1) values, specimens were weighed with an accuracy of 0.001 g using a precision analytical balance instrument (Adam Equipment 4 digits precision weighing balance, Adam Equipment Inc., Oxford, UK). Following that, the samples were placed inside a plastic flask filled with 25 mL of distilled water, kept for seven days, and then incubated for seven days at 37 °C. To determine the mass values of the specimens following immersion, each specimen was then taken out, carefully dried with absorbent paper, and weighed once more (M2). The percentage of solubility was determined using the following equation [39]:

$$\frac{{\text{M}}1-{\text{M}}2}{{\text{M}}1}\times 100\mathrm{\%}$$

where; M1 is the initial mass, and M2 is the final mass of the specimens.

## Statistical analysis

Statistical analysis was conducted by the Statistical Package for Social Sciences (IBM-SPSS version 27.0, New York, NY, USA). Using Kolmogrov-Smirnov and Shapiro–Wilk tests, the data showed a normal distribution. An independent sample t test was used to compare the mean compressive strength (MPa), microhardness (VHN), and solubility (%) for the ZOE (control) and modified ZOE. The significance level was set at *P* ≤ 0.05.

## Results

### XRF results

The chemical composition of E-glass fibers investigated by XRF spectrometry is represented in Table 1. The result of the XRF showed that the major content of the fibers, which was around 52 wt.%, was composed of SiO_2_, and the amounts of MgO and CaO were 19 and 21 wt.%, respectively. The concentration of Al_2_O_3_ was 5 wt.%. Minor contents of K_2_O and B_2_O_3_ were detected, which were 2 and 1 wt.%, respectively.

**Table 1:** Chemical compositions (wt.%) of the E-glass fibers

Chemical Composition	wt. %
SiO_2_	52
Al_2_O_3_	5
B_2_O_3_	1
MgO	19
CaO	21
K_2_O	2

### SEM results

**Fig. 1:**
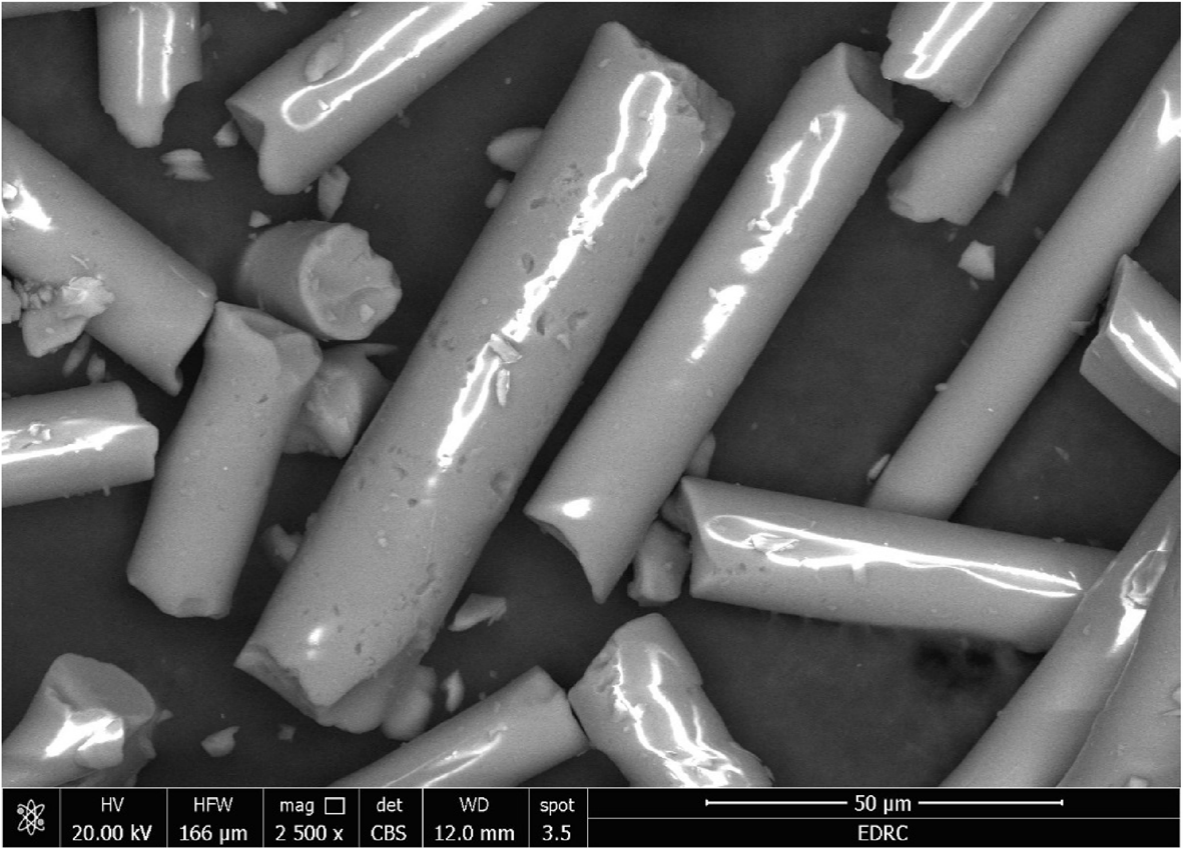
SEM micrograph of E-glass fibers

The SEM micrograph of the E-glass fibers was conducted at 2500 X magnification, as shown in Fig. 1. The SEM images revealed uniform, continuous, slender, straight filament morphology with a long aspect ratio. Moreover, it was clearly seen that the particles’ surface was smooth and dense. Meanwhile, the particle surface was homogenously distributed.

### PSD analysis results

**Fig. 2:**
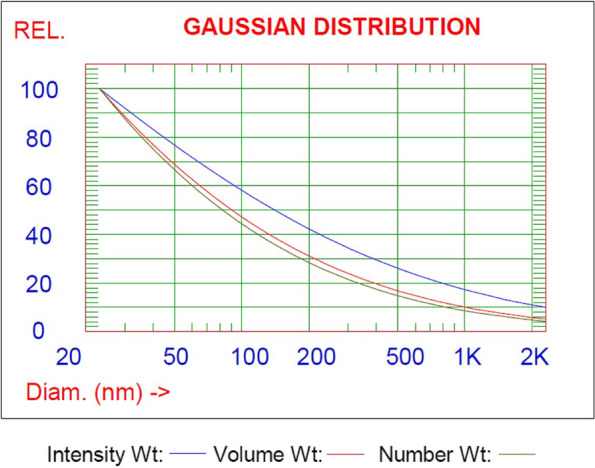
Distribution of E-glass fibers diameter

The PSD analysis of the E-glass fibers was plotted in Fig. 2 and Table 2. The results revealed that 99% of the E-glass average diameter distribution was < 1.94 µm, as represented in Table 3.

**Table 2:** The results of the PSD analysis of E-glass fibers diameter

Volume weighting		Number weighting		Intensity weighting	
Diameter	(%)	Diameter	(%)	Diameter	(%)
0.23 µm	100%	42.5 nm	100%	25.7 nm	100%

**Table 3:** The cumulative intensity-weighted gaussian particle distribution analysis of E-glass fiber diameter

25% of distribution	< 59.4 nm
50% of distribution	< 0.23 µm
75% of distribution	< 0.50 µm
99% of distribution	< 1.94 µm

### Testing results

There were significant differences between the two groups in all the tested properties: compressive strength, microhardness, and solubility. The modified ZOE showed significantly higher compressive strength and microhardness and lower solubility compared to the ZOE (control), as represented in Table 4.

**Table 4:** Mean compressive strength, microhardness, and solubility values between the two groups

Test	ZOE (control)	Modified ZOE	*P* value
Compressive Strength (MPa)	8a ± 0.3	20.3b ± 0.8	0.00001*
Microhardness (VHN)	12.3a ± 0.9	15.8b ± 0.6	0.00001*
Solubility (%)	3.8b ± 0.2	0.7a ± 0.1	0.00001*

Different small letters in the same row are significant difference, * Denotes significant difference as *P* ≤ 0.05

## Discussion

ZOE cement is one of the most frequently implemented temporary materials in dentistry [41]. They vary greatly in their properties depending on their usage. It has been used as a temporary filling, primary pulp canal obturating material, periodontal dressing, and intermediate and thermal insulating base in restorative operations. Generally, these are weak cements. However, they have been demonstrated to permit a sedative, palliative, and antibacterial effect on exposed dentin and are the least irritating of all dental types of cement [41, 42]. Since the majority of the ZOE cement is based on weak ZnO powder, it is believed that the mechanical properties of ZOE could be strengthened by the addition of more potent fillers [3].

There are several elements that affect dental material durability, including its mechanical properties, surface microhardness, and solubility [43, 44]. Surface microhardness generally denotes a material's resistance to abrasion and plastic deformation [45, 46]. The stability, biocompatibility, and longevity of restorative materials are significantly impacted by their solubility [40, 44, 47].

Incorporation of E-glass fibers into dental materials to reinforce them is becoming more and more popular since they have strength and biocompatibility comparable to dental tissues and a very pleasing aesthetic [30, 43]. Moreover, E-glass fibers have superior surface microhardness and limited solubility and degradation [48, 49]. Since then, few studies in the literature have been conducted to improve the properties of ZOE cement. The current study was carried out to improve the compressive strength, surface microhardness, and solubility of the ZOE cement by incorporating 10 wt.% silane-treated E-glass fiber. The silane treatment on the fiber has a significant enhancement in the adhesion between the fiber and matrix, which is a crucial factor in improving the mechanical properties of the composite [50, 51]. The percentage of 10% filler incorporation was established after a pilot study to obtain the most achievable properties.

XRF analysis is a standard and reliable technique that is frequently used for glass chemical investigation, mostly due to its speed and affordability [52, 53]. XRF analysis was performed to determine the quantitative composition of the E-glass fiber fillers prior to their use. The results of XRF analysis revealed that the major content of the fibers was silica-based, ranging from 50–60 wt.% SiO_2_, and comprised a variety of other oxides of magnesium, calcium, boron, sodium, aluminum, and potassium, which are frequently employed for polymer reinforcement. [54]. The results of the XRF conformed to the chemical composition and content of the commonly used reinforcing E-glass fibers [55].

SEM imaging is a perfect tool for examining how evenly and uniformly glass fibers are distributed [56]. The SEM analysis was done to determine the distribution, orientation, aspect ratio, and morphology of the fibers [36]. The results obtained from SEM images denote continuous, slender, long fibers, which is an important factor in induced strengthening effects. It was noted that the mechanical properties of continuous long glass fiber-reinforced composites are superior to those of short glass fiber [57, 58].

To optimize the use of filler particles, particle size and distribution analysis are accurate and crucial methods. It is regarded as a precise technique for determining the particles' maximum and mean diameters [59]. The cumulative intensity-weighted gaussian particle distribution results of E-glass fiber diameter showed that 99.9% of the average diameter distribution of E-glass was < 1.94 µm, which may lead to homogeneous and dense powder packing [60].

The null hypothesis was rejected as the modification of the ZOE by the incorporation of 10 wt.% silane-treated E-glass fibers produced a significant effect on the compressive strength, surface microhardness, and solubility values compared to the ZOE control group.

Based on the current study's results, the compressive strength of dental ZOE was significantly improved upon adding 10 wt.% E-glass fiber fillers. The anticipated strengthening effect of incorporating E-glass fiber fillers may be the cause of this finding [33, 34]. Moreover, the silane treatment of the fibers may be responsible for the suitable adhesion of the fillers to the composite [50, 51]. In addition, the employment of continuous, long E-glass fibers may cause an improvement in the compressive strength [57, 58].

These results are in accordance with previous studies conducted by Ferreira et al., who concluded that the addition of at least 10% niobophosphate bioactive glass to ZOE enhances its compressive strength compared to unmodified ZOE cement [37].

Furthermore, the findings of the compressive strength test met the minimum 5 MPa value requirement specified in ISO standard 3107 for class II materials used as bases and temporary restorations (ISO 3107:2022). The readings showed a clear correlation with the concentration found in the particles, indicating that the particles are providing the cement with a mechanical support [38].

The results suggest that there is more resistance to abrasion and distortion when the indenter is loaded into the modified groups. The presence of a hard E-glass fiber fillers phase within the matrix, which acts as the strongest reinforcement, may be the cause of the increased surface microhardness observed in the modified groups [61, 62]. The reinforcements withstand the applied stresses, which raise the hardness and reduce plastic deformation [61]. The inclusion of glass fibers has been evident in improving the surface microhardness [62]. The results of the study come in agreement with another study conducted by Thipperudrappa et al., who concluded that the incorporation of ZnO nanofiller into E-glass fiber epoxy composites improved their surface microhardness values [63].

Regarding the solubility percentages, the results obtained from the modified groups showed a reduction in solubility; this finding may be explained by the limited solubility of the incorporated E-glass fibers [48, 64].

The experimental situations did not perfectly mimic the clinical ones, which is a limitation of the current study. A limitation of this study is that the amount of eugenol liquid used for the reinforced cement with E-glass being the same as in the unmodified one. Moreover, microstructural examination is needed to detect any voids not included in the current study. Further studies are recommended to investigate the possible effects of incorporating E-glass fibers into ZOE with different aspect ratios, fibers orientations, directions, and concentrations. Moreover, more investigation is required to assess the rheological properties of the modified materials, and to study their other mechanical properties in addition to surface roughness. It is recommended to perform further thermal, tribological, and microstructural examinations to get intensive information about the surface qualities, thermal behavior, and formation of voids of the modified cements. In addition, the cytotoxicity of the modified cement should be examined in further study. Further investigations are required to assess the sealing ability of the modified ZOE by E-glass when applied in the tooth structure.

## Conclusions

The innovatively modified ZOE with 10 wt.% silane-treated E-glass fiber fillers had the opportunity to be used as permanent filling materials with enhanced compressive strength, surface microhardness, and solubility compared to the unmodified ZOE. The salinization of the fillers into the matrix seems to be of great importance. The reinforced cement could be used as a permanent cement for dental purposes.

## Data Availability

The data that support the findings of this study are available from the corresponding author upon reasonable request.

## Abbreviations

ZOE: Zinc-oxide eugenol
ZnO: Zinc-oxide
wt.%: Weight percent
SEM: Scanning electron microscope
PSD: Particle size distribution
DLS: Light scattering technique
XRF: X-ray fluorescence
MREC: Medical Research Ethical Committee
NRC: National Research Centre
ISO: International Organization for Standardization
VH: Vickers hardness
VHN: Vickers microhardness number
MPa: Megapascal
